# Clinical experience of SIB-IMRT in anal cancer and selective literature review

**DOI:** 10.1186/1748-717X-9-199

**Published:** 2014-09-08

**Authors:** Stefan Janssen, Christoph Glanzmann, Peter Bauerfeind, Sonja Stieb, Gabriela Studer, Michelle Brown, Oliver Riesterer

**Affiliations:** Departments of Radiation Oncology, University Hospital Zurich, Rämistrasse 100, CH-8091 Zurich, Switzerland; Clinic of Gastroenterology and Hepatology, University Hospital Zurich, Zurich, Switzerland

**Keywords:** SIB-IMRT, Anal cancer, Definitive radio-chemotherapy

## Abstract

**Purpose:**

To evaluate feasibility and outcome of our institutional SIB-IMRT schedule in patients with anal cancer and to selectively review the literature on different SIB-IMRT schedules.

**Patients and methods:**

Between 01/08-06/13 25 patients with biopsy proven squamous cell anal cancer were treated in our institution with IMRT. Radiotherapy was delivered in two series using a SIB-IMRT schedule of 45 Gy/1.8 Gy to the primary tumor and adjacent pelvic lymph nodes and 38 Gy/1.52 Gy to elective nodes followed by an IMRT boost of 7×2 Gy = 14 Gy to the primary tumor and involved nodes (cumulative prescription dose: 59 Gy).

**Results:**

Mean follow-up was 20 months (range: 4-68). The 2-year-local control, colostomy-free survival, distant metastases-free survival and overall survival rates were 92%, 92%, 92%, and 88%, respectively. Grade 3 acute skin toxicity was observed in 6 patients (24%). No high grade gastrointestinal or urinary acute toxicity occurred. Four patients required more than one day of treatment interruption due to acute toxicity. No grade 3 or higher late sequelae were observed.

**Conclusion:**

We present our institutional SIB-IMRT experience treating patients with anal cancer in two series using moderate single doses from 1.5-2.0 Gy. Our results, in terms of loco-regional outcome and toxicity, were comparable to other studies. The incidence of treatment interruptions was very low. Therefore this schedule appears to be safe for clinical use.

## Introduction

Anal cancer is rare with an incidence of only 1-2/100.000 [1]. Since Nigro et al. observed high rates of response in the neoadjuvant setting, organ preserving definitive radio-chemotherapy has been the standard of care [2]. Randomized phase III trials confirmed superiority of concurrent radio-chemotherapy using 5-fluorouracil (5-FU) and mitomycin C (MMC) compared to radiotherapy alone [3, 4] or platinum-based radio-chemotherapy [4].

The loco-regional control rates of patients treated with radio-chemotherapy in randomized phase III trials ranged from 61% to 84% after 5 years [3–5]. In RTOG 98-11 major acute G3 or higher toxicities were skin reactions (49%), hematologic effects (61%), and gastrointestinal toxicities (37%). Major late G3 or higher toxicities were skin reactions (3%) and gastrointestinal problems (3%) [5]. So far, all prospectively randomized phase III studies on radiation therapy of anal carcinoma used 3-D conformal radiotherapy [3–5]. In recent years, several multi- and single institution studies demonstrated that intensity-modulated radiotherapy (IMRT) reduced toxicity without compromising outcome [6–12]. The use of highly conformal IMRT for treatment of anal cancer is attractive because of the large treatment volumes adjacent to the small bowel and bladder as well as exposure of the skin in the gluteal fold to high doses. A recent prospective RTOG phase II trial (RTOG 0529) investigated the utility of IMRT in anal cancer. The 2-year loco-regional control rate was 80%. In comparison to the results of RTOG 98-11 the use of IMRT reduced early G3 or higher gastrointestinal toxicity from 36% to 22%, and G3 or higher skin toxicity from 47% to 20% [13]. However, until long term control rates become available, concerns remain regarding potential compromise of tumor control rates using more conformal radiotherapy. IMRT can be delivered in several consecutive series using cone-down boost technique but also offers the possibility to deliver treatment giving different doses to different target volumes at the same time (simultaneously integrated boost (SIB) or “dose painting”). The optimal technique of IMRT with or without SIB is still under debate, because the use of a SIB requires altered fractionation schedules that might compromise tumor control, if too low dose per fraction is used, or increase toxicity if doses above 2 Gy fractions are used. Up to date no standard SIB IMRT schedule has been established.

Here we present the outcome of 25 anal cancer patients treated consistently with our institutional SIB-IMRT schedule. Additionally we undertook a selective literature review on clinical studies investigating different SIB-IMRT schedules for treatment of patients with anal cancer.

## Patients and methods

### Patients

From 01/2008 to 06/2013 25 patients with biopsy proven squamous cell anal cancer were included in the analysis (Table 1).

**Table 1:** **Patient related parameters**

Age	
Mean age (years)	61
Range	41-90
Gender	
Male	16 (64%)
Female	9 (36%)
Tumor stage	
T1	3 (12%)
T2	12 (48%)
T3	8 (32%)
T4	2 (8%)
Nodal stage	
N0	13 (52%)
N1	6 (24%)
N2	4 (16%)
N3	2 (8%)
Distant metastases	
M0	25 (100%)
M1	0 (0%)

All patients alive at the time of analysis were contacted by telephone (S.J.) or were recently seen in our department for regular follow-up visits. Additional information was obtained from general practitioner and attending specialist. Patient and treatment related parameters are summarized in Tables 1 and 2.

**Table 2:** **Treatment related parameters**

Postoperative IMRT	2 (8%)
Definitive IMRT	23 (82%)
RT dose	
**Series 1** SIB: 45 Gy SIB	25 (100%)
IMRT (25x1.8/1.52 Gy)	
**Series 2** Boost:	
7x2 = 14 Gy (total: 59 Gy)	20 (80%)
7x2.1 = 14.7 Gy (total: 59.7 Gy)	1 (4%)
8x1.8 = 14.4 Gy (total: 59.4 Gy)	1 (4%)
6x1.8 = 9.8 Gy (total: 55.8 Gy)	1 (4%)
5x1.8 = 9 Gy (total: 54 Gy)	2 (8%)
Treatment breaks >1 day	4 (16%)
Concomitant chemotherapy (5-FU and MMC)	21 (84%)
Mean treatment volumes (ccm, range)	
GTV	33.2 (6-103)
PTV38 Gy	1042.9 (248-3222)
PTV45 Gy	1483.3 (482-2874)
PTV59 Gy	335.1 (56-666)

IMRT: intensity modulated radiotherapy, SIB: simultaneously integrated boost, GTV: gross tumor volume, PTV: planning target volume.

### SIB-IMRT

All patients underwent a planning-CT in supine position. Gross tumor volume (GTV) was defined as macroscopic disease (primary with/without lymph nodes). Elective node regions such as inguinal and iliacal, obturator and presacral lymph nodes were delineated as clinical target volumes (CTV). The GTV was expanded by at least 2 cm and the CTV by 1 cm for the planning target volume (PTV). A second planning CT was performed around 40 Gy for definition of the boost volume. The boost PTV usually contained a margin of at least 2 cm from the primary tumor and 1-2 cm from the nodal GTV.

SIB-IMRT was performed using the following institutional standard schedule:

1^st^ series: 45 Gy in 1.8 Gy single doses to the pelvis, involved lymph nodes (PTV1) and lymph nodes at high risk for microscopic disease (usually lower internal and external iliac nodes) and 38 Gy in 1.52 Gy single doses to elective nodes (PTV2). Elective nodes were mainly uninvolved inguinal and upper internal iliac nodes.

**Figure 1:**
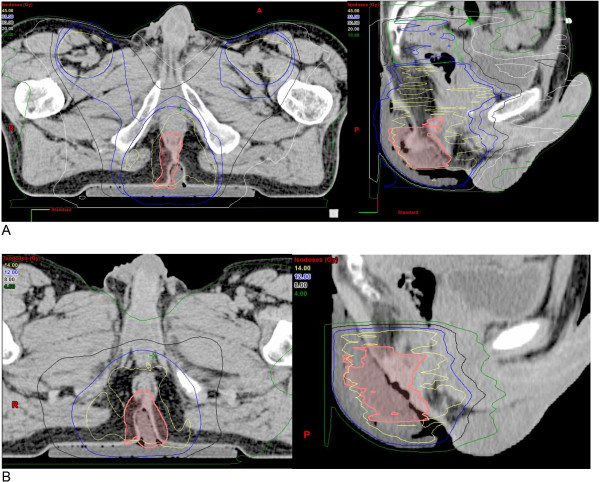
**SIB-IMRT plan. (A)** SIB-IMRT plan for a 47-year old male anal cancer patient (cT2cN0M0). Light green: 38 Gy, blue: 45 Gy pelvis field. **(B)** IMRT boost volume, second series to the tumor. Yellow: 14 Gy. Pink: GTV.

2^nd^ series: IMRT boost of 7×2 = 14 Gy given to the primary tumor and lymph node metastases (total prescription dose: 59 Gy). In 2 patients with low risk tumors the boost was reduced on an individual basis to a total dose of 54 Gy (Figure 1).

For treatment planning, the dose was normalized to the mean dose in PTV1. For intensity optimization, the prescribed dose encompassed at least 95% of the PTV. Additionally, no more than 2% of any PTV received >110% of its prescribed dose, whereas no more than 1% of any PTV received <93% of the prescribed dose. Irradiation was delivered with four to five coplanar beam angles by a 6-MV dynamic MLC system (Varian Medical Systems, Palo Alto, CA) using a sliding window technique, or using volumetric modulated arc technique (VMAT, n = 19, since 10/2010).

### Systemic therapy

Systemic therapy preferably consisted of concomitant 5-fluorouracil (5-FU) day 1-5 (750 mg/m^2^, maximum absolute dose: 1500 mg) and mitomycin C (MMC) day 1 (10 mg/m^2^, maximum absolute dose: 20 mg) in week one and five (n = 21). In 5 patients, chemotherapy was reduced or stopped after the first cycle due to blood count changes and/or reduction of general condition. Four patients did not receive any systemic therapy due to age and/or reduced Karnofsky performance score (n = 2) or postoperative setting (n = 2).

### Assessment of toxicity

Acute and late toxicity was assessed according to the Common Terminology Criteria for Adverse Events (CTCAE) version 4.0.

### Statistical analysis

Kaplan Meier survival analyses were performed using SPSS version 21.

Analysis was approved by the ethics committee of the Zurich university hospital.

## Results

### Disease control

**Figure 2:**
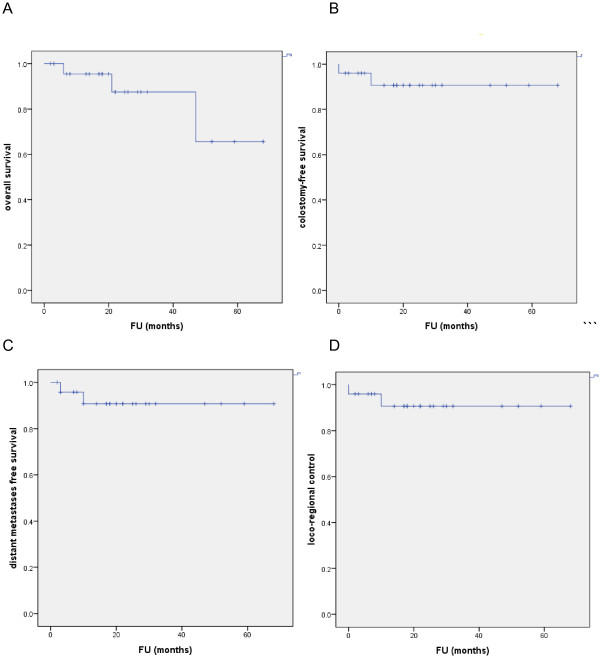
**Survival curves. A-D**: Overall survival, colostomy-free survival, distant metastases free survival, loco-regional control, FU=follow-up.

The mean follow-up time was 20 months (range: 4-68). The 2-year local control, disease free survival, colostomy-free survival, metastasis-free survival and overall survival rates were 92%, 92%, 92%, 92% and 88%, respectively (Figure 2).

At the time of analysis, 3/25 (12%) patients were dead. In two cases this was tumor related due to primary tumor progression (n = 1) or distant metastases (n = 1), whilst the third patient died from a progressive lung cancer.

An 86 year old patient with a stage T2N0G3 cancer treated with chemoradiation to 59 Gy developed a local recurrence 10 months after RT completion. A salvage colostomy was carried out. The patient is still alive and free of disease. A 61 year old female patient with a stage T3N0G2 cancer treated with chemoradiation to 59 Gy suffered from tumor persistence and died 21 months after RT completion due to reduced general condition/local and distant tumor progression. One patient developed lung metastases three months after completion of radio-chemotherapy and died due to distant tumor progression after 3 months.

### Early radiation induced side effects

Grade 3 acute skin toxicity was observed in 6 patients (24%) and grade 3 acute hematologic toxicity in 4 patients (16%). No other grade 3 or higher acute toxicity was seen. If only patients with radio-chemotherapy were considered acute grade 3 skin and hematologic toxicity was seen in 5/21 (24%) and 4/21 (19%), respectively. 15 patients had no treatment breaks. Six patients had one single day of therapy interruption, 2 patients 2 days and one patient missed 3 and 4 days, respectively.

### Late tolerance

Late toxicity was reported as grade I proctitis in one patient 18 months post IMRT completion, one patient suffered from intermittent diarrhea 14 months after completion of radio-chemotherapy and another patient had a slight insufficiency of sphincter muscle (22 months after IMRT). No grade 3 or higher late sequelae were observed.

## Discussion

Patients with anal cancer have been treated with organ preserving radio-chemotherapy since the findings of Nigro et al. [2]. Radio-chemotherapy is superior to radiotherapy alone [3]. Efforts to replace MMC by cisplatin have not been beneficial in terms of tumor control [5]. As loco-regional control rates of up to 84% at 5 years are acceptable, a major focus is reduction of treatment related side effects which have been remarkably high with acute ≥ grade 3 hematologic toxicity of 26%/61%, skin reaction of 48%/49% and gastrointestinal toxicity of 16%/37% in UK anal cancer trial (ACT II) and RTOG 98-11, respectively [5, 14].

IMRT is a technique to deliver radiotherapy with modulated beams holding the chance to deliver highly conformal doses to the target volume while sparing organs at risk. Several series beginning in the early 2000`s reported their experiences with IMRT in anal cancer treatment.

### Side effects

Studies comparing results of patients treated with IMRT and 3-D plans identified reduced major acute and late non-hematological toxicities without a compromise in outcome [6, 7, 9]. As an example, Chuong et al. found a reduction of acute ≥ G3 non-hematologic toxicity of almost 40% (p = <.0001). Menkarios et al. and Brooks et al. showed a significantly reduced dose to small bowel, bladder and genitalia compared to 3-D planning [10, 11]. The study groups of Salama and Kachnic evaluated IMRT in comparison with historic standards and confirmed significant reduction of acute and late toxicity [8, 13]. In the RTOG 0529 phase II IMRT study, there was a significant reduction of acute grade 2+ hematologic (73% vs. 85%), grade 3+ gastrointestinal (21% vs. 36%) and grade 3+ dermatologic adverse events (23% vs. 49%) in comparison to the results of RTOG-98-11 [15]. In terms of efficacy and toxicity our results also compare favorably with the randomized trials using 3-D-conformal techniques and are comparable to recent IMRT studies (Table 3). Before the IMRT era anal cancer patients in our institution were treated with 3-D conformal 45 Gy to the pelvis plus 14.4 Gy photon boost or 14 Gy high dose rate brachytherapy with Ir192 (in 7 fractions). In published results of 81 patients overall acute grade 3 or 4 toxicity of 43% (photon boost) and 15% (brachytherapy boost) was observed. Chronic toxicity occurred in 19% (brachytherapy boost) and 30% (photon boost) [16]. In the current IMRT series we observed acute grade 3 skin toxicity in only 24% of patients. Only 3 patients complained about low grade late toxicity and no grade 3 or higher late sequelae were seen. Response rates in both series are comparable.

**Table 3:** **Different SIB-IMRT schedules in treatment of anal cancer**

Study/year	Patients	Mean FU (months)	SIB-IMRT dose levels (prescription total dose/single dose)	Number of series	Range of SIB-single dose	DFS	LRC	CFS	OS	Acute ≥ grade 3 toxicity
Menkarios 2007	5	*	Concept: 2 dose levels (SIB):	1-2	1.5-1.8 Gy	*	*	*	*	*
			49.5/1.5 Gy							
			59.4/1.8 Gy							
			or							
			2 series (45/1.8 and 59.4/1.8 Gy)							
Salama 2007	53	14.5	PTV: 32-60.9 Gy (median: 51.5 Gy)	1-2	1.65-2.0 Gy		84%/1.5y	84%/1.5y	93%/1.5y	GI: 15%
										Skin: 38%
			ENI: 30.6-45 Gy (median: 45 Gy)							Hematologic: 59%
			Concept: 3 dose levels:							
			41.25/1.65 Gy,45/1.8, 50/2.0 (+/-boost)							
Vieillot 2010	10	*	Concept: 2 dose levels:	1	1.5-1.8 Gy	*	*	*	*	*
			49.5/1.5 Gy							
			59.4/1.8 Gy							
Call 2011	34	22	PTV: 48.6-57.6 Gy (median: 50.4 Gy)	1	1.28-2.25 Gy	80%/3y			87%/3y	Not reported
			ENI: 38-45 Gy							
			No standard concept							
Barzan 2011	29	32	Concept: 3 dose levels:	2	1.6-1.8 Gy		92/3y	91/3y	88%/3y	GI: 7%
										Skin: 21%
			40/1.6 Gy							
			45/1.8 Gy							Hematologic: 21%
			+boost 5.4 Gy (T1/2), 9-14.4 Gy (T3/4)							
Kachnic 2012	43	24	Concept: T-stage based SIB (2 dose levels)	1	1.5-1.8 Gy		95%/2y	94%/2y	92%/2y	GI: 7%
										Skin: 10%
			T2N0: 42/1.5 Gy ENI, 50.4/1.8 Gy to PTV							Hematologic: 51%
			T3-4N0-3: 45/1.5 Gy ENI,							
			50.4/1.68 Gy to lymph nodes<3cm							
			54/1.8 Gy to PTV and lymph nodes>3cm							
Deenen 2012	18	28	49.5/1.5 Gy ENI	1-2	1.5-1.8 Gy		83%/2y			GI: 0%,
			59.4/1.8 for PTV							Skin: 50%
			Boost 5.4/1.8 Gy for macroscopic residual tumor after 5 weeks							Hematologic: 0%
Mitchell 2013	65	19	PTV: 50-58.8 Gy (median: 54 Gy)	1	1.62-2.0 Gy	86%/2y			96%/2y	GI: 9%
										Skin: 17%
			ENI: 40.5-50.4 Gy (median: 45 Gy)							Hematologic: 3%
			Concept: T-stage based SIB (2 dose levels):							
			T1: 50/2 Gy, 43/1.72 Gy							
			T2: 54/2, 45/1.67 Gy							
			T3/4: 58/2 , 47/1.62 Gy							
Present study	25	20	45/1.8 Gy to PTV	2	1.52-2 Gy	92%/2y	92%/2y	92%/2y	88%/2y	GI: 0%,
			38/1.52 Gy ENI							Skin: 23%
			14/2 Gy boost to PTV in second series (total: 59 Gy)							Hematologic: 16%

*comparison of different plans, *ENI* = Elective node irradiation, *FU* = Follow-up, *GI* = Gastrointestinal, *PTV* = Planning target volume.

### Treatment breaks

In comparison to other published results, the treatment breaks in our patient cohort were considerably low with only 8% of patients in need of more than 1 day of treatment break due to acute toxicity. These data reflect the good tolerability of our approach. Salama et al. found breaks in 41.5% of patients, lasting a median of 4 days [8]. Bazan et al. reported on 34.5% treatment breaks with IMRT, which was significantly less than with 3-D planning (88%) [7]. In RTOG 98-11, treatment breaks were reported in 62% and were mainly required secondary to radiation toxicities. One reason for low acute skin toxicity in our patients could be sophisticated nursing with specially trained nursing staff provided in our outpatient clinic. As treatment breaks are shown to compromise treatment outcome in anal cancer patients [17, 18], this is an important finding.

### SIB-IMRT in anal cancer patients

IMRT, in principle, enables delivery of different doses to different target volumes (e.g. pelvic and inguinal lymph nodes) in the same treatment session (SIB, “dose painting”). This leads to a shortening of the treatment course. In addition, only one or two physic plans have to be generated and approved. There is still concern regarding the use of a SIB technique in anal cancer because more conformal dose distribution and altered dose per fraction might compromise outcome and/or increase toxicity.

As summarized in Table 3, several different SIB IMRT schedules are described in the literature. In some studies, the total dose varied according to T stage [12, 13]. Most studies administered SIB in only one series whereas 4 studies conducted 2 series [7, 8, 10, 19]. SIB dose per fraction ranged from 1.28 Gy to 2.25 Gy and total doses from 30.6 Gy to 59.4 Gy. When we implemented IMRT in anal cancer, we decided to deliver SIB-IMRT carefully in 2 series with single fractions of maximally 2 Gy, because we did not want to risk increased toxicity. In comparison to many US centers and the RTOG trials, in which 45 Gy/ 50.4 Gy were given to T1/T2 and 55-59 Gy/54 Gy to T3/4 tumors (RTOG 98-11, RTOG 0529), we usually give 59 Gy regardless of T stage with very few exceptions for very small primary tumors.

In our opinion, the concept of two series has two major advantages: Firstly, the single dose to elective nodes is not below 1.52 Gy to 1.8 Gy and the dose to the GTV can be kept at 2 Gy fractions. Secondly, the boost holds the chance to escalate doses in 2.0 Gy single doses on an individual basis. Limitations of our study are its retrospective design, the limited number of patients and short follow-up. However, we present an easy to use SIB-IMRT schedule and our clinical data and experience suggest that this schedule is safe and efficacious when compared to other published series.

## Conclusion

We present a new SIB-IMRT schedule to treat patients with anal cancer in two series using moderate single doses from 1.5-2.0 Gy with a total dose of 59 Gy in combination with 5FU/MMC. Our results, in terms of loco-regional control and toxicity, are comparable to the results of other studies. Remarkably, the incidence of treatment interruptions was very low. Therefore this schedule appears to be safe and favorable for clinical use. More prospective studies on SIB-IMRT schedules are needed in order to define a standard.

### Informed consent

Written informed consent was obtained from the patient for the publication of this report and any accompanying images.
